# Foodborne Pathogen Survival in Commercial *Aloreña de Málaga* Table Olive Packaging

**DOI:** 10.3389/fmicb.2018.02471

**Published:** 2018-10-16

**Authors:** Verónica Romero-Gil, Eduardo Medina, Antonio Garrido-Fernández, Francisco Noé Arroyo-López

**Affiliations:** ^1^Department of Food Biotechnology, Instituto de la Grasa (CSIC), University Campus Pablo de Olavide, Seville, Spain; ^2^Regulatory Council of Protected Designation of Origen Aloreña de Málaga Table Olives, Málaga, Spain

**Keywords:** challenge tests, food safety, phenolic compounds, survival models, antimicrobials

## Abstract

This study presents an approach to determine the survival of diverse foodborne pathogens (*Escherichia coli, Staphylococcus aureus, Listeria monocytogenes*, and *Salmonella enterica*) in three *Aloreña de Málaga* table olive commercial presentations (fresh green, traditional, and cured olives). The microbial survival in this green natural table olive speciality was fit using a log-linear regression model implemented in GInaFIT. The contents of sugars, phenolic compounds, additives, salt, pH, and levels of autochthonous microorganisms differed among presentations and affected the survival of microorganisms. The inoculated initial populations of pathogens (7–8 log_10_ CFU/mL brine) decreased rapidly and, 48 h after inoculation, their counts were always below the detection limit (<1.3 log_10_ CFU/mL), except for *S. aureus* in the fresh green presentation which was ∼ 5.0 log_10_ CFU/mL. The highest maximum death rates (*k_max_*) and lowest periods for 4 log_10_ reductions (*4Dr*) were observed in cured olives but decreased and increased, respectively, from the traditional to the fresh green presentations. *L. monocytogenes* and *S. aureus* were the most resistant species. The multivariate analysis showed that high concentrations of compounds released from the olives (sugars and phenols) were positively associated to *4Dr* and negatively to *k_max_*. Conversely, the presence of preservatives reduced *4Dr*. This study, executed in commercial products, pointed out that packaged table olives are adverse habitats for foodborne pathogens with their effects being presentation dependent. The survival of *S. aureus* was particularly long in green fresh *Aloreña de Málaga* table olives packaged without preservatives; therefore, its changes in this presentation still requires further in-deep research.

## Introduction

Table olives are one of the most traditional fermented vegetables in the Mediterranean basin, with a worldwide production of around 2.5 million tons per year (International Olive Oil Council [IOC], 2016). In addition to natural/chemical treatments, the diverse microbial growth that takes place during storage/fermentation makes them edible and is responsible for their appreciated organoleptic properties. The control of such microbiota is essential during processing and, notably, after packaging to guarantee the stability and safety of the final product, especially in those presentations commercialized without heat treatments (Garrido-Fernández et al., 1997).

The *Aloreña de Málaga* cultivar is processed as natural cracked green olives under a Spanish Protected Designation of Origin (PDO) recognized by the European Union (DOUE, 2012). Their peculiar characteristics are related to the production area (climate, edaphology, and geographical location) and presentation, which make their products different from other green table olives. *Aloreña de Málaga* usually contains low-to-moderate concentrations of oleuropein (the main bitter compound of olives), and for this reason, is not subjected to lye debittering. This speciality is frequently seasoned with fennel, thyme, garlic, and pepper, making it rich in aroma. To preserve their typical green aspect and organoleptic characteristics, *Aloreña de Málaga* fruits are not usually stabilized by heat treatment. Therefore, safety issues related to the hygienic conditions and studies on the prevalence of foodborne pathogens in these table olives (ready to eat) are of particular interest for risk assessment during storage/shelf life.

The PDO regulation includes 3 different commercial presentations (from now on just presentations) of *Aloreña de Málaga* table olives (López-López and Garrido-Fernández, 2006), which are:

(i) Fresh green *Aloreña de Málaga* olives. The product is characterized by the immediate cracking after harvesting. Then, the fruits are brined in a 10–11% NaCl solution (in 220 L volume plastic drums), where they remain only for 3 days, after which the partially debittered olives are seasoned and packaged.
(ii) Traditional *Aloreña de Málaga* olives. The fruits are also cracked and brined (10–11% NaCl, in 200 L volume plastic drums) after harvesting, but the olives are stored in the NaCl solution for, at least, 20 days. During this period, the fruits undergo a partial fermentation, which intensity depends on the storage time. Then, the olives are seasoned and packaged according to demand.
(iii) Cured *Aloreña de Málaga* olives. In this case, the harvested fruits are placed directly in brine (5–6% NaCl, in 10,000 L volume vessels) where they undergo a full fermentation for a minimum of 90 days. Then, the olives are progressively cracked, seasoned and packaged, according to demand.

The predominant microorganisms in table olives are lactic acid bacteria (LAB) and yeasts; however, the presence of pathogenic bacteria such as *Escherichia coli, Listeria monocytogenes, Staphylococcus aureus*, and *Salmonella* spp. in table olives from different origins has been reported (Spyropoulou et al., 2001; Caggia et al., 2004; Pereira et al., 2008; RASFF Portal, 2012; Argyri et al., 2013; Grounta et al., 2013; Medina et al., 2013; Tataridou and Kotzekidou, 2015). However, the relationship between the physicochemical and microbiological characteristics of olive packaging and the behavior of foodborne pathogens in the different olive products has been scarcely studied. In a previous study carried out in our laboratory (Medina et al., 2016), the survival of the foodborne pathogenic species *E. coli, S. aureus, L. monocytogenes*, and *S. enterica* in a model system based on sterile brines obtained from fermented *Aloreña de Málaga* fruits was determined. The bactericidal effect was related to the presence of certain phenolic compounds. However, the response in such model systems may differ substantially from that in commercial products since the presence of fruits may continue the release of nutrients into the brine after packaging and substantially modify the physicochemical environment. Furthermore, the presence of preservatives commonly added in table olive packing can also influence the survival.

The objective of the present approach was to investigate the prevalence of these pathogenic species in the different presentations of the *Aloreña de Málaga* speciality, taking into account the possible effect of the food matrix and the use of preservatives. Multivariate analysis was used to associate the pathogens’ behavior with the environmental characteristics of the packages (physicochemical parameters, polyphenols, sugars, additives and indigenous microbiota).

## Materials and Methods

### Foodborne Pathogens

In this survey, 4 strains of each pathogenic species *L. monocytogenes, S. aureus, E. coli* and *S. enterica* were obtained from the Spanish Type Culture Collection (CECT, Valencia, Spain). All of them are related to human food poisoning and included in the biosafety level 2. *L. monocytogenes* CECT 4031^T^ (isolated from rabbit), CECT 4032 (soft cheese), CECT 5366 (human origin) and CECT 7467 (poultry) were cultured at 37°C in Brain Heart Infusion (BHI, Oxoid LTD, Basingstoke, England) with or without agar (15 g/L). *S. aureus* CECT 86^T^ (isolated from human pleural fluid), CECT 239 (human lesion), CECT 240 (human lesion) and CECT 976 (ham); *S. enterica* sv. Typhimurium CECT 722^T^ (natural environment), CECT 443 (human), CECT 4156 (chicken alimentary tract), and *S. enterica* sv. Enteritidis CECT 4300 (unknown); *E. coli* CECT 434 (clinical isolate) and the strains with serotype O157:H7 CECT 4267, CECT 4782 and CECT 5947 (isolated from human feces) were all cultured at 37°C in nutrient broth prepared with 5 g/L “Lab-Lemco” powder (Oxoid), 10 g/L of Neutralized bacteriological peptone (Oxoid), 5 g/L NaCl, and 15 g/L of agar in the case of solid medium (pH 7.2). All pathogen strains were maintained at -80°C in the adequate culture broths with 20 g/L glycerol until their use.

### Commercial Presentation Packaging

This work was carried out with commercial packages of seasoned cracked *Aloreña de Málaga* olives. The packages were collected in the 2015/2016 season from 4 different industries located in the Guadalhorce Valley (Málaga, Spain) and included the 3 PDO *Aloreña de Málaga* presentations: fresh green (Fresh), traditional (Trad), and cured (CUR-A and CUR-B from different industries). Industry transparent PET (polyethylen terephthalate) packages were filled with 0.5 or 0.7 kg of fruits, seasoning material (a mixture of diced garlic, pepper strips, and small pieces of fennel and thyme) and 0.3 or 0.5 L of cover brine for both fresh/traditional and cured olives, respectively. Twelve different containers (intended for further inoculation) were obtained for each presentation and industry, making a total of 48 table olive packaging. In parallel, 3 packages from each industry were randomly chosen to determine their composition in phenolic, oleosidic compounds, and reducing sugars in brines as described below.

### Physicochemical Analysis

Salt, pH, titratable and combined acidity were determined according to the protocols described by Garrido-Fernández et al. (1997).

The analysis of phenolic compounds was carried out as described elsewhere (Medina et al., 2007). Briefly, a mixture of 250 μL of brine, 250 μL of internal standard (2 mM syringic acid) and 500 μL of deionised water was filtered through a 0.2 μm pore size nylon filter, and an aliquot (20 μL) was injected into the liquid chromatography. The chromatographic system consisted of a Waters 717 plus autosampler, a Waters 600 E pump, a Waters column heater module, and a Waters 996 photodiode array detector operated with Empower 2.0 software (Waters Inc.). A 25 cm × 4.6 mm i.d., 5 μm, Spherisorb ODS-2 (Waters Inc.) column, at a flow rate of 1 mL/min and a temperature of 35°C, was used in all experiments. Separation was achieved by gradient elution using (A) water (pH 2.5 adjusted with 0.15% phosphoric acid) and (B) methanol. The gradient used was described by Medina et al. (2007). The wavelengths selected for phenolic and oleosidic compounds were 280 and 240 nm, respectively (see **Supplementary Figures S1, S2**). The evaluation of each compound was performed using a regression curve with the corresponding standard. Hydroxytyrosol, oleuropein and verbascoside were purchased from Extrasynthese SA (Genay, France). Tyrosol, caffeic and p-coumaric acids were from Sigma Chemical Co. (St. Louis, MO, United States). Secologanoside, and comselogoside were quantified using the response factors of caffeic acid, and p-coumaric acid, respectively. Hydroxytyrosol-4-glucoside, secoxyloganin, oleoside 11-methyl ester and the dialdehydic form of decarboxymethyl elenolic acid free (EDA) or linked to hydroxytyrosol (HyEDA) were obtained using a HPLC preparative system (Medina et al., 2007). The analyses were performed in duplicate.

Individual reducing sugars (glucose, fructose, sucrose, and mannitol) and organic acids (lactic and acetic) were determined by HPLC according to the methods developed by Rodríguez-Gómez et al. (2012). For the analysis of sugars, 0.5 mL of liquid and 1.5 mL of sorbitol internal standard (1 g/L, w/v) were put into contact with 1 g IRA 120 (Hƥ-form, 16e45 mesh, Fluka) and 1 g IRA 96 (free base, 20e50 mesh, Fluka) resins. After 1 h of contact, 0.5–1.0 mL was centrifuged. The clarified liquid was used for analysis using an Aminex HPX-87C carbohydrate analysis column (BioRad Labs, Hercules, CA, United States) held at 85°C. Deionized water was used as eluent at 0.6 mL/min. For the organic acids, a Spherisorb ODS-2 (5 mm, 250 4.6 mm, Waters Inc.) column with deionized water (pH adjusted to 2.3 with phosphoric acid) as mobile phase was used. The flow rate was 1.2 mL/min. Samples (0.5 mL) were diluted (1:1) with deionized water, centrifuged at 11,600 *g* for 5 min and an aliquot of 20 μL was injected into the chromatograph. The system was composed of a Water 2690 Alliance (which includes a pump, column, heater and auto-sampler modules). The detections were achieved using a Water 410 Differential Refractometer (**Supplementary Figure S3**). Quantification was achieved by comparison of the peak areas with the corresponding standards. The analyses were performed in duplicate.

Information about the additive levels used in each packaging (potassium sorbate, sodium benzoate, citric and ascorbic acid levels) were kindly provided by the industry.

### Microbiological Analysis

The population levels of the autochthonous microorganisms in the different presentations of *Aloreña de Málaga* table olive packaging were also determined before inoculation. Cover brine samples were diluted as needed in a 0.85% NaCl sterile solution and plated using a Spiral System model dwScientific (Dow Whitley Scientific Limited, England) on appropriate media. *Enterobacteriaceae* were counted on Violet Red Bile agar supplemented with Glucose (1%) (VRBG) (Merck, Darmstadt, Germany), LAB were spread onto de Man-Rogosa, and Sharpe (MRS) agar (Oxoid) supplemented with 0.02% sodium azide (Sigma, St. Luis, MO, United States), and yeasts were grown on a yeast–malt–peptone–glucose medium (YM) agar (Difco, Becton and Dickinson Company, Sparks, MD, United States) supplemented with oxytetracycline and gentamicin sulfate (0.005%, wt/vol) as selective agents for yeasts. The plates were incubated at 30°C for 24 (*Enterobacteriaceae)* or 72 h (yeasts and LAB, respectively). Then, plates were counted using a Flash & Go (IUL, Barcelona, Spain) image analysis system. Brine counts were expressed as log_10_ CFU/mL.

### Pathogen Challenge Assays

Before inoculation, each foodborne pathogen strain was cultured in the respective culturing broth medium with a previous pre-adaptation phase where NaCl (15 g/L) and pH (5.5) were modified to adapt the microorganisms to the olive brines. The cocktail of each pathogenic species was prepared by mixing equal broth quantities of their diverse strains. Overnight cultures (12 h) in the early stationary phase were centrifuged, and pellets were washed in a sterilized saline solution (9 g/L NaCl) and centrifuged again. Ten per cent of brine volumes were withdrawn from the olive packaging in sterile conditions and used to re-suspend the cell pellets. After mixing, the suspension of the strain cocktail of each species was returned as inoculum. The volumes were calculated to obtain ca. 8 log CFU/mL of each species as initial inoculum in the olive packaging. An enumeration of the inoculated cells to confirm the initial population was done in triplicate, using the appropriate medium.

The challenge tests were performed in triplicate at room temperature (20 ± 2°C), obtaining the average of 3 independent inoculations. Samples from each olive packaging were removed at different times (0, 1.5, 6, 24, and 48 h), diluted if necessary in 1.0 g/L peptone, and plated to count pathogen cultivable cells in appropriate media. Baird-Parker agar (Oxoid) was used for *S. aureus*, MacConkey agar w/sorbitol (Laboratorios Conda, Madrid, Spain) for *E. coli*, Xylose Lysine Desoxycholate Agar (Laboratorios Conda) for *S. enterica* and Palcam *Listeria* agar base supplemented with selective supplement (Laboratorios Conda) for *L. monocytogenes*. The responses of the different pathogen cocktails in the 3 types of *Aloreña de Málaga* table olive packaging were fitted using the log-linear regression model (Bigelow and Esty, 1920), included in the GInaFIT 1.6 software excel fitting tool (Geeraerd et al., 2005). For modeling, data after the first value below the detection limit (≤1.3 log_10_ CFU/mL) were censored. The linear regression model used had the following expression:

$$\begin{array}{c} {\mathrm{Log}}_{\mathrm{10}}(N_{t})\;=\;\log_{\mathrm{10}}(N_{0})-k_{max}{}^{*}\mathrm{t/ln}(\mathrm{10}) \end{array}$$

where the parameters were: *N_t_*, the microbial population at time *t* (log_10_ CFU/mL), *N_0_*, initial microbial population (log_10_ CFU/mL), and *k_max_*, maximum death rate (h^-1^). The software GInaFIT 1.6 also allows the estimation of *4Dr* (time in hours for a reduction of 4 log_10_ from *N_0_*).

### Statistical Analysis

An analysis of variance (ANOVA) was performed using the factorial ANOVA module of Statistica 7.1 software package (StatSoft Inc, Tulsa, OK, United States) to determine statistical differences among the response of the several foodborne pathogen cocktails in the packaging from the 4 industries (but only 3 presentations). *Post hoc* comparisons were achieved using the Scheffé test, which is the most flexible and conservative (that is, produces the widest confidence intervals) since it corrects α-values for all pair-wise or straightforward comparisons of means, but also for all multiple comparisons of means as well (Box et al., 1978).

The data (a matrix consisting of the concentrations of phenols, sugars, organic acids, preservatives, initial LAB and yeast populations, and the parameters deduced from the model fit to the pathogen survival) were also subjected to hierarchical cluster analysis (centered data, Euclidean distance, Ward’s method) to disclose the dissimilarities among the diverse treatments, replicates within them, and study the profiles of the presentations. The effect of the variables on the parameters obtained from the fit inhibition models was also studied by Partial Least Square regression (PLS), using centered and standardized data. PLS combines features from the Principal Component Analysis and multiple regression, which application is specifically appropriate in the case of correlated variables. The first step is the selection of latent variables able to explain the maximum proportion of independent variable variances and as much as possible of the dependent variable set. The extracted factors (latent variables) also have the best predictive power of the dependent variable. The advantage of this statistical technique over classic regression is the available charts that describe the data structure. Thanks to the correlation and loadings, and score plots, the relationship among the variables (the exploratory, dependents, and between both of them) and the proximity of samples and dataset structure are studied (Abdi, 2007). The number of latent variables was retained automatically. The performance of the fit equations obtained from the inhibition response (*4Dr* and *k_max_*) as a function of independent variables (phenolic compounds, organic acids, preservatives, and initial microbiota) for the cocktail of each pathogen was checked by cross-validation. The multivariate analyses were performed using XLSTAT v.2015.4.01.20116 software (Addinsoft, Paris, France) and Minitab 15 v. 15.1.20.0 (Minitab Inc., State College, PA, United States).

## Results

### Analysis of Commercial Presentations

The physicochemical characteristics of the 3 industrial olive presentations were analyzed. The 3 *Aloreña de Málaga* table olive presentations showed clear differences among them (**Table 1**); the highest total sugar content was found in the fresh green (9.6 g/L), followed by traditional (5.7 g/L), and cured olives (0.42 and 2.03 g/L in samples A and B, respectively), being glucose and fructose the major components. The highest total phenolic compound was also observed in the fresh green presentation (8.9 mM) and decreased from traditional (3.9 mM) to cured olives (3.4 and 2.9 mM in samples A and B, respectively). There were also differences among the presentations in the concentration of specific polyphenols. Secoxyloganin, oleuropein, and hydroxytyrosol 4-Glucoside (Hy 4-Glu) were the 3 most abundant compounds in fresh green while their levels were lower in the traditional and cured presentations. On the contrary, hydroxytyrosol (Hy) was the phenol with the highest concentration in traditional and cured olives, followed by tyrosol (Ty). EDA (the dialdehydic form of decarboxymethyl elenolic acid) was never detected, while HyEDA (EDA linked to Hy) was only found (at 0.22 mM) in the fresh green elaboration. The pH, titratable acidity, and salt concentration levels also differed considerably among presentations, ranging from 3.80 (CUR-B) to 4.76 (Fresh) in the case of pH, 1.50 (CUR-A) to 5.43 (Trad) g/L (expressed as lactic acid) for titratable acidity, and 47.30 (CUR-A) to 61.30 (Fresh) g/L for NaCl. The range for combined acidity, 0.02 – 0.05 mEq/L, was tight. Traditional and cured *Aloreña de Málaga* olives were initially acidified with citric (0.9 – 2.0 g/L) and ascorbic (0.5 – 1.0 g/L) acids and were preserved using different concentrations of potassium sorbate (1.0 – 1.8 g/L) and sodium benzoate (1.0 – 2.20 g/L), while none of them was added to the organic presentation (fresh green).

**Table 1:** Physicochemical characteristics and additives levels of the different *Aloreña de Málaga* commercial packaging brines used in the present study.

Parameter	Compound (Abbreviation)	Fresh	Trad	CUR-A	CUR-B
Salt (g/L)	NaCl	61.30 (0.09)^c^	56.20 (0.03)^b^	47.30 (0.04)^a^	55.10 (0.02)^b^
pH	[H+]	4.76 (0.05)^b^	3.95 (0.03)^a^	4.75 (0.02)^b^	3.80 (0.01)^a^
Titratable acidity (g/L)	Expressed as lactic acid (TA)	2.37 (0.01)^b^	5.43 (0.01)^d^	1.50 (0.01)^a^	2.90 (0.01)^c^
Combined acidity (mEq/L)	– (CA)	0.05 (0.01)^a^	0.05 (0.01)^a^	0.04 (0.01)^a^	0.02 (0.01)^b^
Sugars (g/L)	Glucose (Glu)	6.541 (0.196)^d^	3.686 (0.751)^c^	0.105 (0.016)^a^	1.172 (0.017)^b^
	Fructose (Fru)	1.937 (0.078)^c^	1.092 (0.471)^c^	0.167 (0.004)^a^	0.329 (0.030)^b^
	Sucrose (Sac)	0.453 (0.011)^c^	0.085 (0.028)^b^	ND	0.033 (0.005)^a^
	Mannitol (Man)	0.634 (0.006)^d^	0.799 (0.028)^c^	0.150 (0.007)^a^	0.500 (0.013)^b^
	Total sugars (TS)	9.565 (0.211)^d^	5.662 (0.886)^c^	0.422 (0.018)^a^	2.034 (0.037)^b^
Phenolic compounds (mM)	Hydroxytyrosol (Hy)	1.116 (0.094)^a^	1.856 (0.321)^ab^	2.604 (0.171)^b^	1.440 (0.034)^a^
	Hy 4-Glucoside (Hy4Glu)	1.267 (0.174)^b^	0.151 (0.074)^a^	ND	ND
	Tyrosol (Ty)	0.423 (0.090)^a^	0.495 (0.118)^a^	0.811 (0.054)^b^	0.446 (0.075)^a^
	*p-*coumaric acid (pCum)	0.030 (0.012)^a^	0.021 (0.010)^a^	ND	ND
	Verbascoside (Verb)	0.193 (0.034)^ab^	0.222 (0.146)^b^	ND	0.093 (0.011)^a^
	HyEDA	0.225 (0.001)	ND	ND	ND
	Oleuropein (Ole)	1.513 (0.102)^b^	0.095 (0.011)^a^	ND	0.116 (0.026)^a^
	Comselogoside (Coms)	0.031 (0.005)^b^	0.015 (0.004)^a^	ND	0.011 (0.004)^a^
	EDA	ND	ND	ND	ND
	Secoxyloganin (Secox)	2.810 (0.080)^b^	0.635 (0.413)^a^	ND	0.446 (0.023)^a^
	Secologanoside (Seclog)	0.651 (0.019)^b^	0.198 (0.134)^a^	ND	0.131 (0.005)^a^
	Oleoside 11-methyl ester (Oleo11)	0.603 (0.010)^c^	0.248 (0.010)^b^	0.006 (0.005)^a^	0.281 (0.016)^b^
	Total phenolic compounds (TPh)	8.862 (0.256)^a^	3.936 (0.561)^a^	3.421(0.179)^a^	2.964 (0.092)^b^
Additives (g/L)	Potassium sorbate (KS)	NA	1.0	1.8	1.5
	Sodium benzoate (NaB)	NA	1.0	2.2	1.5
	Ascorbic acid (AA)	NA	1.0	0.5	0.5
	Citric acid (CAc)	NA	2.0	0.9	1.5

*Standard deviations obtained from triplicated experiments in parentheses. EDA, dialdehydic form of decarboxymethyl elenolic acid. HyEDA, EDA linked to hydroxytyrosol. ND, not detected. NA, not added. Additive concentrations refer to the levels in brine reported by the industries. Values followed by different superscript letters, within the same row, are significantly different according to the Scheffé posthoc comparison test.*

Regarding the initial presence of autochthonous microorganisms in brine, *Enterobacteriaceae* were always below the detection limit (<1.3 log_10_ CFU/mL) while LAB and yeasts were found at statistically significant different populations (*p* < 0.05). The highest LAB counts were obtained in fresh green presentation (6.7 ± 0.2 log_10_ CFU/mL), followed by cured olives from industry A (6.4 ± 0.1 log_10_ CFU/mL), cured olives from industry B (2.4 ± 1.5 log_10_ CFU/mL), and the traditional packaging (1.33 ± 0.04 log_10_ CFU/mL). Yeast populations followed a similar trend (4.6 ± 1.2, 4.2 ± 1.2, 2.1 ± 1.1, and 1.8 ± 0.8 log_10_ CFU/mL, respectively). Therefore, the highest initial microbial population levels were observed in the fresh green, followed by the cured and the traditional presentations.

The considerable differences in pH, salt, acids, sugar, phenolic compounds, preservatives and autochthonous microbial populations lead to different environmental characteristics among the *Aloreña de Málaga* presentations which, presumably, should lead to different survival profiles of pathogens.

### Survival of the Foodborne Pathogens in Olive Presentations

**FIGURE 1:**
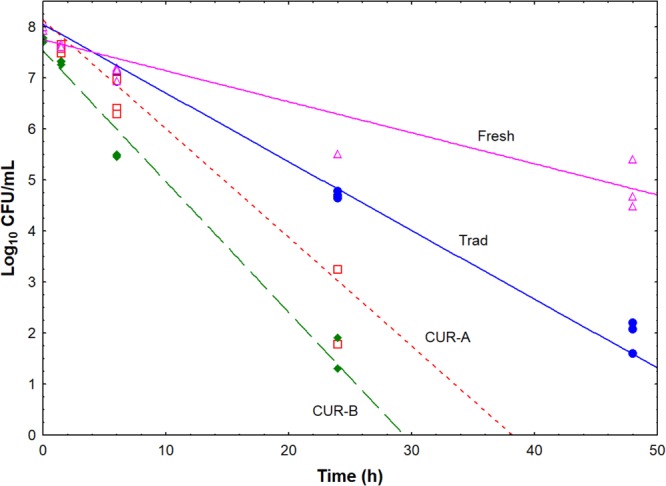
Fit of the log-linear regression model to the survival data of the *S. aureus* cocktail in the 3 commercial presentations. Trad, traditional *Aloreña de Málaga* presentation; Fresh, fresh green presentation; CUR-A and CUR-B refer to the two industries which elaborated the cured presentations.

The independent inoculation of the 4 pathogen cocktails in the 3 *Aloreña de Málaga* presentations (performed in triplicate) led to a total of 16 different treatments and 48 cases. **Supplementary Tables S1–S4** shows the survival data for all foodborne pathogens assayed in the different commercial presentations. The fit of the population changes of each pathogen cocktail *vs.* time, using a log-linear regression model (GInaFIT Excel tool), was always good, with an *R*^2^ usually above 0.97. The model assumes a linear inactivation of pathogens (*k_max_*) from the initial population level (*N_0_*) to below the detection limit (<1.3 log_10_ CFU/mL). The responses and fit of the *S. aureus* cocktail in the 3 presentations (including two industries for cured fruits) are reported as an example in **Figure 1**; the cured olives (CUR-B and CUR-A treatments) showed the highest *k_max_* (slope), followed by the traditional and the fresh green presentations (**Figure 1**). The other pathogenic species followed similar trends. Therefore, results consistently indicated that the fresh green presentation had the lowest inhibitory effect on pathogens’ survival.

The fitted models estimated *N_0_* values (**Table 2**) ranging from 8.5 (*E. coli* in cured olives from industry A) to 7.0 log_10_ CFU/mL (*L. monocytogenes* in cured olives from industry B). The *k_max_* oscillated from 0.14 (*S. aureus* in the fresh green) to 0.67 h^-1^ (*E. coli* in cured olives from industry A), while the *4Dr* parameter ranged from 14 (*E. coli* in cured olives from industry A) to >48 h (*S. aureus* in the fresh green presentation). The standard deviations of the model parameters (obtained from triplicate experiments) were, in general, low. The *k_max_* and *4Dr* values in the cured olives were higher and lower respectively than those found in fresh green and traditional presentations.

**Table 2:** Parameters obtained using a Log-linear regression model (Bigelow and Esty, 1920) implemented in GinaFit to the survival data of the foodborne pathogen cocktails in the *Aloreña de Málaga* commercial packaging.

Pathogen/Parameter	Fresh	Trad	CUR-A	CUR-B
*E. coli*				
N_0_ (log_10_ cfu/mL)	7.7 (0.1)^b^	8.4 (0.2)^a^	8.49 (0.05)^a^	7.12 (0.33)^b^
*k_max_* (h^-1^)	0.5 (0.1)^a^	0.6 (0.2)^a^	0.67 (0.03)^a^	0.59 (0.02)^a^
*4Dr* (h)	21 (7)^a^	19 (9)^a^	13.9 (0.6)^a^	15.9 (0.4)^a^
*L. monocytogenes*				
N_0_ (log_10_ cfu/mL)	7.95 (0.03)^c^	8.11 (0.03)^a^	8.20 (0.05)^a^	7.04 (0.01)^b^
*k_max_* (h^-1^)	0.30 (0.04)^a^	0.29 (0.02)^a^	0.65 (0.01)^b^	0.54 (0.00)^c^
*4Dr* (h)	31 (4)^a^	31 (1)^a^	14.40 (0.00)^b^	17.1 (0.1)^b^
*S. aureus*				
N_0_ (log_10_ cfu/mL)	7.75 (0.09)^b^	8.05 (0.03)^a^	8.1 (0.2)^a^	7.51 (0.02)^b^
*k_max_* (h^-1^)	0.14 (0.02)^a^	0.30 (0.02)^a,b^	0.5 (0.2)^b^	0.59 (0.03)^b,c^
*4Dr* (h)	>48^c^	31(1)^a^	19 (7)^b^	15.8 (0.8)^b^
*S. enterica*				
N_0_ (log_10_ cfu/mL)	7.7 (0.2)^b^	8.15 (0.04)^a^	8.1 (0.2)^a^	7.52 (0.02)^c^
*k_max_* (h^-1^)	0.32 (0.01)^c^	0.65 (0.00)^a^	0.64 (0.02)^a^	0.61 (0.01)^b^
*4Dr* (h)	29 (1)^c^	14.2 (0.1)^a^	14.2 (0.5)^a^	15.12 (0.01)^a^

*Standard deviations obtained from triplicate experiments in parentheses. *N_*0*_*, initial microbial population; *4Dr*, time for a reduction of 4 log_*10*_ from *N_*0*_*_;_*k_*max*_*, maximum death rate. Values followed by different superscript letters, within the same row, are significantly different according to the Scheffé posthoc comparison test.*

**FIGURE 2:**
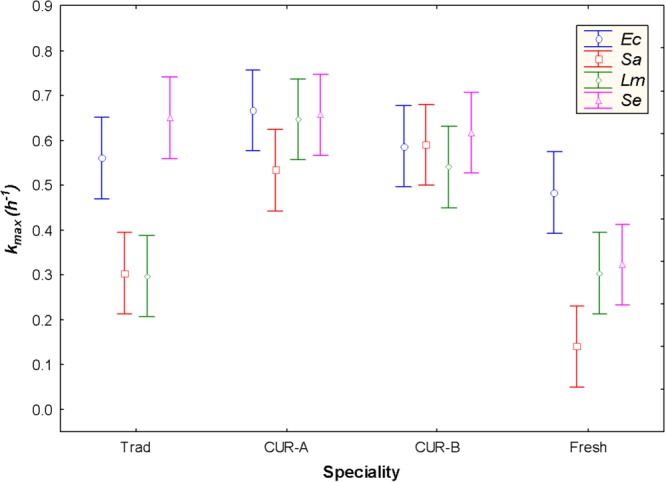
Graphical representation of the factorial ANOVA analysis carried out for the inhibition parameters *k_max_* (maximum death rate) obtained with the log-linear regression model to the survival data of the foodborne pathogens in the different presentations (*4Dr*, time for 4 log_10_ reduction, followed a similar but opposed trend). *Sa, Ec, Lm* and *Se* stand for the *S. aureus, E. coli, L. monocytogenes*, and *S. enterica* cocktails, respectively. Trad, traditional *Aloreña de Málaga* presentation; Fresh, fresh green presentation; CUR-A and CUR-B refer to the two industries which elaborated the cured presentations. Vertical bars denote 0.95 confidence limits.

The ANOVA analysis showed that the main effects and the interactions of categorical variables (pathogen cocktails and *Aloreña de Málaga* presentation) on the inhibition parameters were statistically significant (*p* < 0.05) (except for *N_0_*), indicating significant differences among, at least, two presentations. Notably, the response of foodborne pathogens was different in traditional and fresh green *Aloreña de Málaga* olives. *S. aureus* was the most resistant microorganism in the fresh green presentation (highest *4Dr* and lowest *k_max_*), while *L. monocytogenes* and *S. aureus* showed great resistance in the traditional product (**Table 2**). Therefore, the behavior of *S. aureus* in the fresh green presentation requires a further in-deep study. Conversely, the survival of the 4 foodborne pathogen species had a very similar behavior in cured olives (industries A and B), with non-statistically different values for *k_max_* and *4Dr* (**Table 2**). These comparisons are visualized for *k_max_* in **Figure 2**. According to these results, fresh green and traditional *Aloreña de Málaga* presentations had lower inhibitory effects and longer survival periods for the two gram-positive than for the two gram-negative pathogenic bacteria assayed.

### Multivariate Analysis

The number of variables able to affect the survival of pathogens in packaged olives are numerous and, subsequently, disclosing their specific roles is complex. Furthermore, the concentrations of most compounds in the 3 presentations are proportional or had similar levels when intentionally added. Hence, the presence of strong correlation among the influential variables is habitual, particularly within and between phenols and sugars (see **Supplementary Table S5**) and support the convenience of subjecting the data to multivariate analysis. Also, the presence of salt and preservatives were positively and negatively related to the contents of organic compounds in brine (**Supplementary Table S5**). Moreover, the values of *4Dr* and *k_ma_*_x_ (except *k_max_* for *E. coli*) were significantly (positive, and negative, respectively) related to most phenols and sugars; that is, they apparently promote the pathogens’ survival (high *4Dr* and low *k_max_*) (see **Supplementary Table S5**). However, the inactivation mechanism for *E. coli* could be different since its *k_max_* was not related to phenols.

**FIGURE 3:**
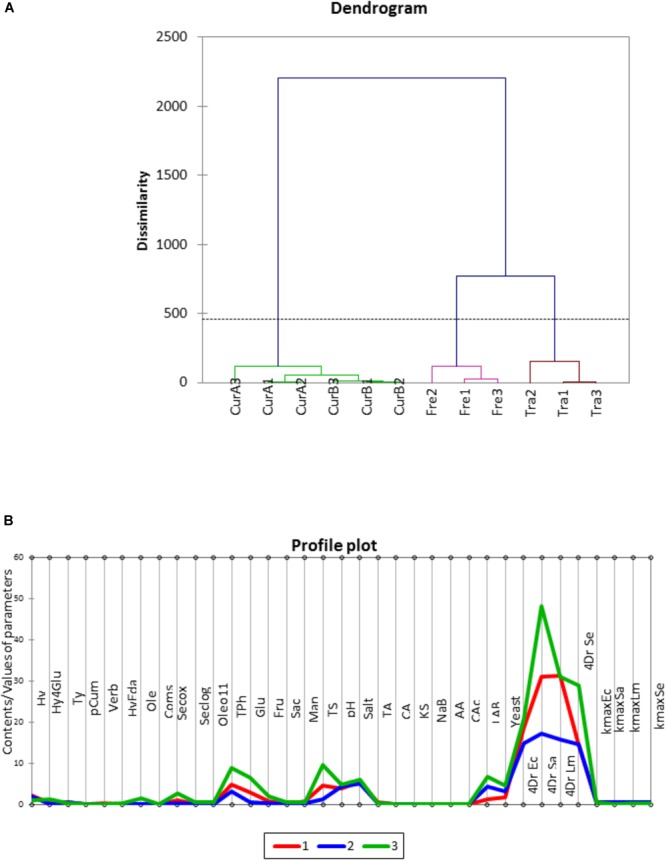
Cluster analysis of treatments, and their replicates, based on polyphenols, sugars, pH, salt, organic acids, preservatives, and fit parameters **(A)**, and their profiles **(B)**. Trad, traditional *Aloreña de Málaga* presentation; Fresh, fresh green presentation; CUR-A and CUR-B refer to the two industries which elaborated the cured presentations. Lines 1, 2 and 3 represents the grouped clusters. See **Table 1** for the rest of abbreviations.

**FIGURE 4:**
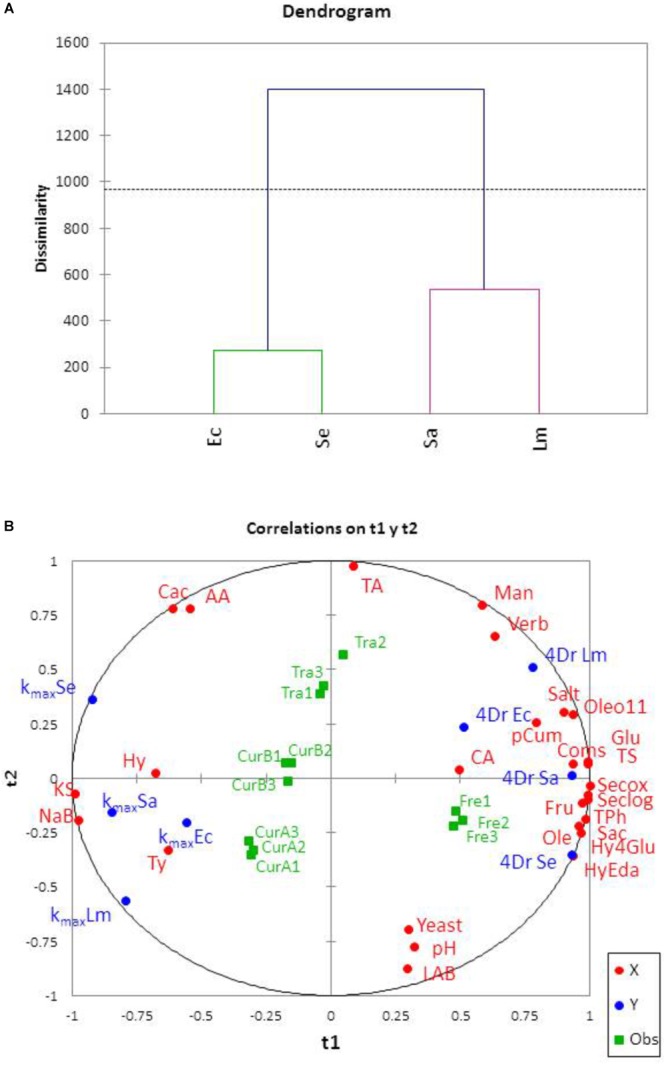
Cluster analysis of pathogen’s behavior based on the fit parameters **(A)** and correlation wheel of the variables studied on the plane of the first two PLS components **(B)**. *4Dr*, time in hours for a reduction of 4 log_10_ from *N_0_*_;_ kmax, maximum death rate (h^-1^); *Ec, Sa, Lm*, and *Se* stand for the different cocktails assayed, *E. coli, S. aureus, L. monocytogenes*, and *S. enterica*, respectively. LAB, lactic acid bacteria. See **Table 1** for the meaning of the rest of abbreviations.

Application of hierarchical clustering led to the segregation of treatments into 3 groups: freshly processed, traditional, and the cured olives (both CUR-A and CUR-B together) (**Figure 3A**). The characteristic profiles of clusters (**Figure 3B**) indicated marked differences among them in the concentrations of total phenol, total sugar, and the *4Dr* values of the pathogens. After clustering, the pathogens’ survival in the different presentations (**Figure 4A**) showed similarities between *E. coli* and *S. enterica* as well as between *S. aureus* and *L. monocytogenes* but both groups were, in turn, clearly different. Such segregation can be useful for designing specific strategies to control each cluster which are, in turn, simultaneously efficient against both groups of pathogens.

The important correlations between independent variables prevent their use for modeling the pathogens’ survival by just multiple regressions. Therefore, the task was achieved by applying PLS regression to the centered values of the variables. The analysis was performed using XLSTAT and Minitab. There were retained 4 latent variables which explained 95% (*R*^2^X = 0.974) and 93% (*R*^2^Y = 0.931) variances of the independent and dependent variable, respectively and had a global Q^2^cum = 0.750. Most of the independent variables were strongly related to the *t1* axis (**Figure 4B**) while a reduced number (TA, AA, CAc, Yeast, LAB, and pH) were associated to *t2* (**Figure 4B**). The graph also shows a similar distribution (correlations with the *t1* axis) of the pathogen survival parameters, *4Dr* always on the right and *k_max_* on the left, as expected due to their inverse relationship. Polyphenols (except Hy, Ty) and sugars followed similar trends, presenting positive correlation with the *t1* axis while the preservatives (KS and NaB) were negatively linked to it; that is, the environmental influences of such variables are opposed. In fact, in commercial packages, the presence of high concentrations of most phenols (except Ty and Hy) and sugars are related to high *4Dr* values. In other words, the time to reduce 4 log cycle increased and, subsequently, the *k_max_* decreased as the contents in polyphenols and sugars were higher. Apparently, the pathogens managed to counteract the inhibitory effect of the phenols in brine thanks to the presence of nutrients. The close situation of phenols and sugars in **Figure 4B** was in agreement with the strong correlation found between phenols and sugars (and possible the rest of nutrients released from the olive flesh) due to their similar diffusion rates and, subsequently, concentrations reached in the brines. This explanation of the pathogen’s behavior is also coherent with the situation of presentations (without practical differences between replicates) on the projections (**Figure 4B**): the fresh green presentations on the right of *t1*, the traditional on an intermediate place (their displacement toward the upper part of the graph may be due to their higher values of CAc, AA, and TA) and the cured olives on the left. The distribution is associated with the expected concentrations of phenols and sugars released in them, according to their processing characteristics. This trend is similar to that observed during the storage of directly brined olives, where the presence of increasing concentrations of nutrients (sugar, mineral, or vitamins) may counteract the inhibitory effect of other compounds such as polyphenols and promote the growth of the habitual microbiota (Garrido-Fernández et al., 1997).

Ty and Hy show entirely opposed linkage with the other phenols. However, this association may be caused by their formation by hydrolysis of other compounds located on the right (which decrease implies an increase in Ty and Hy concentrations) and, therefore, the role of Hy and Ty on *k_max_* and *4Dr* should be considered with caution. The position of the pH and initial yeast and lactic acid bacteria are strongly (negatively) correlated to *t2* and opposed to CAc, AA, and TA (positively linked to *t2*), indicating an opposed effect between both groups of variables. Interestingly, the presence of preservatives is highly (and negatively) correlated to *t1* which, in turn, means high and positive association with *k_max_* and, on the contrary, opposed linkage to *4Dr*. Then, the addition of preservatives to the cover brines decreased the pathogen’s survival due to their systematic associations with *k_max_* (positive) and *4Dr* (negative) in all pathogens. Those presentations with higher contents in KS and NaB (cured) showed higher *k_max_* and lower *4Dr* values than presentations with no or low added preservatives (traditional and fresh green). Therefore, at least under the packaging conditions of this study, the presence of preservatives was, apparently, more determinant for a rapid decrease in the pathogen populations than the composition of the cover brines (polyphenols, sugars, pH, or total acidity). The results, then, emphasize the essential role that preservatives may have on the pathogen survival in real industrial packaging.

The values of the parameters used for the fit evaluation (**Table 3**) showed that the models were appropriate, and the *4Dr* and *k_max_* could be estimated as a function of the environmental variables, using the coefficients obtained from the PLS analysis (see **Supplementary Table S6**). The standardized coefficients (which do not depend on the size of the measures) are appropriate parameters for indicating the variables that most contribute to the estimation (**Table 4**). For example, the positive contributors for *4Dr* of *E. coli* were Coms (1.585), Hy (1.142), pCum (0.863), KS (0.732), while the negative were Hy4Glu (-0.561), HyEDA (-0.659), and LAB (-0.815). In the case of *4Dr* of *S. aureus*, the most outstanding influential variables were pH (0.634), KS (0.753), and NaB (0.527), all of them positive, while the influences of the variables with negative signs were reduced. The contributions to the other *4Dr* or *k_max_* survival parameters may be evaluated similarly. To emphasize, the peculiar behavior of *S. enterica* which coefficient profile was different from the rest.

**Table 3:** Goodness of fit statistics of the models deduced for the estimation of the inhibition curve parameters, according to the pathogen.

Fit parameter	*4Dr*				*k_max_*			
	*Ec*	*Sa*	*Lm*	*Se*	*Ec*	*Sa*	*Lm*	*Se*
*R*^2^	0.995	0.995	0.997	<0.999	>0.999	0.995	0.999	>0.999
*SD*	1.301	3.032	1.538	0.308	0.004	0.049	0.013	0.005
MSE	0.141	0.766	0.197	0.008	<0.001	<0.001	<0.001	<0.001
RMSE	0.376	0.875	0.444	0.089	0.001	0.014	0.004	0.001

*The number of observations was always 12 and the degree of freedom 1. *4Dr*, time in hours for a reduction of 4 log_*10*_ from *N_*0*_*_*;*_ kmax, maximum death rate (h^-*1*^); *R*^*2*^, determination coefficient; SD, standard deviation; MSE, mean square error; RMSE, root mean square error. *Ec, Sa, Lm*, and *Se* stand for the different microbial cocktails assayed, *E. coli, S. aureus, L. monocytogenes*, and *S. enterica*, respectively.*

**Table 4:** Standardized coefficients for the different mathematical models.

Variable	*4Dr*				*k_max_*			
	*Ec*	*Sa*	*Lm*	*Se*	*Ec*	*Sa*	*Lm*	*Se*
Hy	1.142	0.258	–0.053	–0.180	–1.028	–0.302	0.009	0.183
Hy4Glu	–0.561	0.377	–0.299	–0.036	0.346	–0.462	0.128	0.020
Ty	–0.856	–0.169	0.385	0.065	0.858	0.212	–0.196	–0.046
pCum	0.863	0.147	–0.163	0.031	–0.720	–0.266	0.100	–0.004
Verb	–0.344	–0.105	0.001	0.038	0.131	0.144	–0.003	–0.024
HyEda	–0.659	–0.270	0.485	0.243	0.661	0.359	–0.254	–0.204
Ole	0.432	0.168	0.036	0.062	–0.297	–0.234	–0.041	–0.047
Coms	1.585	0.440	–0.528	–0.259	–1.608	–0.426	0.243	0.182
Secox	0.286	0.238	0.029	0.036	–0.196	–0.331	–0.060	–0.017
Seclog	0.136	0.244	0.012	0.050	–0.048	–0.354	–0.049	–0.029
Oleo11	0.005	–0.099	0.223	0.061	0.013	0.167	–0.158	–0.064
TPh	0.343	0.298	0.006	0.003	–0.283	–0.387	–0.048	0.012
Glu	–0.056	0.351	–0.092	–0.009	0.037	–0.476	–0.008	0.020
Fru	0.047	0.366	–0.188	–0.016	–0.088	–0.483	0.061	0.021
Sac	–0.009	0.056	0.062	0.059	–0.040	–0.029	–0.047	–0.057
Man	0.018	0.277	0.160	–0.051	0.008	–0.394	–0.194	0.080
TS	–0.030	0.344	–0.088	–0.010	0.007	–0.462	–0.010	0.021
pH	0.495	0.634	–0.386	–0.059	–0.381	–0.897	0.201	0.080
Salt	–0.099	–0.313	0.512	0.192	0.287	0.372	–0.307	–0.161
TA	–0.136	0.280	0.126	–0.128	0.093	–0.376	–0.178	0.142
CA	–0.225	–0.421	0.412	0.095	0.281	0.622	–0.202	–0.118
KS	0.732	0.753	–0.543	–0.194	–0.622	–1.132	0.257	0.238
NaB	0.373	0.527	–0.400	–0.152	–0.351	–0.778	0.203	0.181
AA	–0.378	0.294	0.088	–0.104	0.430	–0.484	–0.138	0.137
CAc	0.214	–0.149	0.245	–0.062	–0.175	0.196	–0.171	0.073
LAB	–0.815	–0.077	0.646	0.214	0.669	0.125	–0.367	–0.113
Yeats	0.402	–0.207	–0.356	–0.126	–0.389	0.372	0.208	0.034

*4Dr, time in hours for a reduction of 4 log_*10*_ from *N_*0*_*_;_ kmax, maximum death rate (h^-*1*^); *Ec, Sa, Lm*, and *Se* stand for the different microbial cocktails assayed, *E. coli, S. aureus, L. monocytogenes*, and *S. enterica*, respectively. See **Table 1** for the meaning of the rest of abbreviations. More relevant contributions are printed in bold.*

## Discussion

Table olives, as well as other fermented and acidified vegetable foods, have a long history of microbial safety. Contamination of olives with pathogenic bacteria is not usual and may be accidental due to poor hygiene and unsanitary procedures during cultivation, harvesting and processing, including inadequate cleaning and sanitizing of equipment.

Nevertheless, researchers have recently identified the presence of certain foodborne pathogens such as *L. monocytogenes, E. coli, S. enterica*, and *S. aureus* and studied their survival in several olive matrixes (Spyropoulou et al., 2001; Skandamis and Nychas, 2003; Randazzo et al., 2012; Argyri et al., 2013; Grounta et al., 2013; Medina et al., 2013; Panagou et al., 2013; Medina-Pradas and Arroyo-López, 2015). In general, it was concluded that the olive environment not only prevents the pathogen’s growth but also promotes a fast decrease of their populations. However, Argyri et al. (2013) reported that *L. monocytogenes, E. coli*, and *Salmonella* were inactivated in lye treated green olive Halkidiki *cv.* at low pH (4.2) and high salt concentration (60 g/L) only after 14 days after packaging. Also, a high survival (>24 h) of pathogenic bacteria was observed in natural black Conservolea olives packages (Grounta et al., 2013). These data show marked differences in the survivals of foodborne pathogens, which were presentation dependent, and point out the importance of specific studies for each case.

In previous studies executed in our group by Medina et al. (2016), the survival of foodborne pathogenic species in a brine model system obtained from fermented *Aloreña de Málaga* fruits was presented, and the observed inhibition was related to the effects of certain polyphenols. In this work, the investigation was extended to the real commercial presentations of the same olive speciality where the presence of the olives might modify substantially the environmental characteristics in the cover brine due to the progressive release of diverse (nutrients and inhibitory) compounds into the brine and the presence of additives. Therefore, in contrast to the studies in just brine, this work also takes into consideration the presence of olives (phenols or sugars release) as well as other variables that can be found in marketed olives such as preservatives and autochthonous microorganisms. Furthermore, a multivariate approach was needed to determine the effect of the numerous independent variables involved in the pathogens survival and evaluate their contributions.

Significant differences between pathogen responses in the brine model system and products were noticed. The first dissimilarity was related to the type of mathematical model used to fit the survival of pathogens. In brine, the fit was achieved by a log-linear model with a tail (Geeraerd et al., 2000), while the data were adjusted to a log-linear regression model without a tail (Bigelow and Esty, 1920) in the packaged system. The second difference was related to the survival of pathogens, which was longer in the commercial products. The total inhibition of pathogen in the brine model system was obtained in less than 24 h for all species, with *k_max_* values ranging from 10 (*E. coli*) to 53 h^-1^ (*S. enterica)* and *4Dr* values ranging from 0.36 *(S. aureus* and *L. monocytogenes*) to 0.96 h *(E. coli*). On the contrary, in the commercial packaging, the *k_max_* parameter ranged, depending on presentation, from 0.14 (*S. aureus* in green Fresh olives) to 0.67 h^-1^ (*E. coli* in cured olives), while *4Dr* values ranged from 13.92 (*E. coli* in cured olives) to >48 h (*S. aureus* in fresh green fruits). The lower *k_max_* and higher *4Dr* values obtained in commercial products compared to the brine model system reveal the lower inhibitory power of the former. Apparently, such trend may be associated with the higher presence of nutrients from the olives in the brines of the commercial products. In real packaging, *S. aureus* and *L. monocytogenes* were the more resistant pathogen, especially in traditional and fresh green *Aloreña de Málaga* presentations, while in brine was *E. coli* (Medina et al., 2016). In the specific case of *S. aureus*, albeit a considerable reduction of the initial inoculum level was noticed in both traditional and fresh green olives, the microorganism was still found in both presentations, after 48 h, at 2.0 ± 0.3 log_10_ CFU/mL in traditional, and 5.0 ± 0.5 log_10_ CFU/mL, in fresh green olives. These data are in agreement with those obtained by Argyri et al. (2013) and Grounta et al. (2013) who found *L. monocytogenes* as the most resistant pathogen in the olive environment. Medina et al. (2013) also reported a considerable resistance of *S. aureus* in various olive brines obtained from Hojiblanca, Manzanilla, and Gordal cultivars.

There were also marked differences among brined fresh green, traditional, and cured *Aloreña de Málaga* presentations related to sugar and polyphenol contents derived from the olives. Fresh and traditional presentations are cracked immediately after their arrival to the factory, but they differ on the time elapsed from this operation to packaging: 3 and longer than 20 days storage in brine, respectively. However, the cured olives are brined directly, subjected to lactic acid fermentation, and, cracked and packaged on demand (usually after 6 months). Therefore, due to the diverse processing techniques, the polyphenols and sugars in the studied presentations were in the following progressive lower order: fresh > traditional > cured (although in this case, olives may also contribute with other compounds from the storage/fermentation). Such circumstances provide diverse inhibitory environments for pathogens.

Packaged *Aloreña de Málaga* olives are stabilized by the addition of sodium benzoate and potassium sorbate for preventing the growth of yeasts which may produce gas, cloudy brines and swollen taps (López-López and Garrido-Fernández, 2006). Although the primary function of preservatives is not to inhibit pathogens, it was observed in this work that they may also significantly influence the survival of pathogenic bacteria. Hence, in the olive presentations studied, microorganisms were exposed not only to the inhibitory effects due to polyphenols (Medina et al., 2007, 2013, 2016) and the growth promotion of different concentrations of nutrients but also to a new effect due to the presence of preservatives. As expected, many phenols were not only highly correlated among them but also with the concentrations of glucose and fructose (**Supplementary Table S5**), indicating that, in turn, the levels of both groups of compounds were strongly dependent on the previous period of storage in the holding solutions (fresh and traditional) or fermentation (cured). Most sugars and polyphenols (except Hy and Ty) were also positively and significantly related to salt (**Supplementary Table S5**), possibly due to the more considerable leakage caused as the salt levels were higher. Conversely, the presence of potassium sorbate and sodium benzoate was negatively related to most of them (except pH, TA, AA, CCi) (**Supplementary Table S5**). Apparently, their presence in the brine at packaging may have interfered the leakage of sugars and phenols into the brine.

The relationships deduced from the multivariate analysis have contributed to highlight the substantial differences among the pathogen survival *in vitro* and those in commercial presentations. Medina et al. (2016) showed that the microbial survival in the brine model system was linked to EDA, HyEDA, Hy-4Glu, Ty, and oleoside 11-methyl ester. However, in this work, the multivariate study showed that the microbial survival in real products was mainly associated with the presence of preservatives (highly positively and negatively correlated to *k_max_* and *4Dr*, respectively); however, the total polyphenols and nutrients are oppositely related (high concentrations imply high *4Dr* and low *k_max_* values). Apparently, the presence of olives (with the subsequent leakage of compounds from the flesh into the brine) provided an entirely different environment for pathogen survival. Also, the addition of preservatives introduced new effects that prevailed over those derived from the presence of the olive released compounds.

## Conclusion

*Aloreña de Málaga* table olive packaging provides an adverse habitat for the development of foodborne pathogens, although with considerable differences among presentations. The survival of pathogens in the commercial product was higher compared to the brine model systems. In this work, *S. aureus* was the most resistant species among pathogens, despite its high phenolic content, especially in fresh green olives packaged without preservatives. Multivariate analysis showed that the possible effect of phenols could be counteracted by the simultaneous higher presence of nutrients (sugars) while, on the contrary, the presence of preservatives was associated with a reduction of the pathogens’ survival. Because of the differences in pathogen survival in the presentations, even of the same olive cultivar, each commercial product requires specific challenge tests. According to results, *Aloreña de Málaga* products, once processed, should be distributed after, at least, 48 h packaging to ensure the inhibition of most pathogens in case of hypothetical contamination. More in-depth research on the prevalence of *S. aureus* in fresh green packaged *Aloreña de Malaga* is still required to properly evaluate its survival, particularly in presentations packaged without preservatives.

## Author Contributions

VR-G, EM, and FA-L contributed to the conception and design of the study. AG-F and FA-L organized the database and performed the statistical analysis. EM and FA-L wrote the first draft of the manuscript, while the rest wrote specific sections of the manuscript. All authors contributed to the revision of the manuscript, read and approved the submitted version.

## Conflict of Interest Statement

The authors declare that the research was conducted in the absence of any commercial or financial relationships that could be construed as a potential conflict of interest.
