# Supplementary material for: Foodborne Pathogen Survival in Commercial Aloreña de Málaga Table Olive Packaging
Source: Front Microbiol. 2018 Oct 16;9:2471. doi: 10.3389/fmicb.2018.02471 (PMC6234914; doi:10.3389/fmicb.2018.02471)
Supplement: Supplementary file 1 [file Data_Sheet_1.docx]

**Table S1.** Survival data (log_10_ CFU/mL) for the *E. coli* cocktail in the different *Aloreña de Málaga* commercial elaborations studied.

|  | **Fresh** | | | **Trad** | | | **CUR-A** | | | **CUR-B** | | |
| --- | --- | --- | --- | --- | --- | --- | --- | --- | --- | --- | --- | --- |
| **Time (h)** | Replicated 1 | Replicated 2 | Replicated 3 | Replicated 1 | Replicated 2 | Replicated 3 | Replicated 1 | Replicated 2 | Replicated 3 | Replicated 1 | Replicated 2 | Replicated 3 |
| 0 | 7.88 | 8.04 | 7.95 | 8.77 | 8.70 | 8.60 | 8.71 | 8.73 | 8.68 | 7.81 | 7.68 | 7.75 |
| 1.5 | 7.25 | 7.32 | 7.20 | 7.33 | 7.38 | 7.42 | 7.39 | 7.54 | 7.46 | 7.06 | 7.02 | 6.98 |
| 6 | 6.38 | 6.65 | 5.99 | 7.19 | 7.18 | 7.20 | 7.23 | 7.14 | 7.29 | 5.15 | 4.96 | 3.15 |
| 24 | 1.78 | 3.46 | 2.00 | ≤1.30 | 5.00 | ≤1.30 | 1.78 | ≤1.30 | ≤1.30 | ≤1.30 | ≤1.30 | ≤1.30 |
| 48 | ≤1.30 | ≤1.30 | ≤1.30 | ≤1.30* | ≤1.30 | ≤1.30* | ≤1.30 | ≤1.30* | ≤1.30* | ≤1.30* | ≤1.30* | ≤1.30* |

**Censored data*

**Table S2.** Survival data (log_10_ CFU/mL) for the *S. aureus* cocktail in the different *Aloreña de Málaga* commercial elaborations studied.

|  | **Fresh** | | | **Trad** | | | **CUR-A** | | | **CUR-B** | | |
| --- | --- | --- | --- | --- | --- | --- | --- | --- | --- | --- | --- | --- |
| **Time (h)** | Replicated 1 | Replicated 2 | Replicated 3 | Replicated 1 | Replicated 2 | Replicated 3 | Replicated 1 | Replicated 2 | Replicated 3 | Replicated 1 | Replicated 2 | Replicated 3 |
| 0 | 7.93 | 8.09 | 8.00 | 8.68 | 8.74 | 8.70 | 8.63 | 8.72 | 8.68* | 8.72 | 7.78 | 7.70 |
| 1.5 | 7.67 | 7.67 | 7.61 | 7.57 | 7.53 | 7.52 | 7.48 | 7.65 | 7.57 | 7.56 | 7.31 | 7.33 |
| 6 | 6.94 | 7.20 | 7.15 | 6.97 | 7.08 | 6.99 | 6.40 | 6.29 | 6.97 | 6.93 | 5.47 | 5.49 |
| 24 | 3.42* | 3.88* | 5.51 | 3.24 | 4.70 | 4.78 | 1.78 | 1.78 | 3.24 | 4.64 | 1.90 | ≤1.30 |
| 48 | 5.40 | 4.48 | 4.68 | ≤1.30 | 2.08 | 2.20 | ≤1.30 | ≤1.30 | ≤1.30 | 1.60 | ≤1.30 | ≤1.30 |

**Censored data*

**Table S3.** Survival data (log_10_ CFU/mL) for the *L. monocytogenes* cocktail in the different *Aloreña de Málaga* commercial elaborations studied.

|  | **Fresh** | | | **Trad** | | | **CUR-A** | | | **CUR-B** | | |
| --- | --- | --- | --- | --- | --- | --- | --- | --- | --- | --- | --- | --- |
| **Time (h)** | Replicated 1 | Replicated 2 | Replicated 3 | Replicated 1 | Replicated 2 | Replicated 3 | Replicated 1 | Replicated 2 | Replicated 3 | Replicated 1 | Replicated 2 | Replicated 3 |
| 0 | 7.95 | 7.82 | 7.90 | 7.83 | 7.87 | 7.85 | 7.87 | 7.78 | 7.80 | 6.88 | 7.06 | 7.00 |
| 1.5 | 7.62 | 7.58 | 7.71 | 7.60 | 7.47 | 7.51 | 7.57 | 7.66 | 7.48 | 6.39 | 6.29 | 6.35 |
| 6 | 7.57 | 7.52 | 7.44 | 6.94 | 7.54 | 7.67 | 7.34 | 7.33 | 7.25 | 6.18 | 6.13 | 6.15 |
| 24 | 4.73 | 4.53 | 4.30 | 6.98 | 5.91 | 5.85 | ≤1.30 | ≤1.30 | ≤1.30 | ≤1.30 | ≤1.30 | ≤1.30 |
| 48 | 2.58 | ≤1.30 | ≤1.30 | ≤1.30 | ≤1.30 | ≤1.30 | ≤1.30* | ≤1.30* | ≤1.30* | ≤1.30* | ≤1.30* | ≤1.30* |

**Censored data*

**Table S4.** Survival data (log_10_ CFU/mL) for the *S. enterica* cocktail in the different *Aloreña de Málaga* commercial elaborations studied.

|  | **Fresh** | | | **Trad** | | | **CUR-A** | | | **CUR-B** | | |
| --- | --- | --- | --- | --- | --- | --- | --- | --- | --- | --- | --- | --- |
| **Time (h)** | Replicated 1 | Replicated 2 | Replicated 3 | Replicated 1 | Replicated 2 | Replicated 3 | Replicated 1 | Replicated 2 | Replicated 3 | Replicated 1 | Replicated 2 | Replicated 3 |
| 0 | 8.20 | 8.29 | 8.25 | 8.12 | 8.05 | 8.07 | 9.05 | 7.93 | 8.50 | 8.15 | 8.26 | 8.20 |
| 1.5 | 7.26 | 7.30 | 7.00 | 7.62 | 7.42 | 7.54 | 7.28 | 7.48 | 7.39 | 7.02 | 6.85 | 7.03 |
| 6 | 6.90 | 6.86 | 6.78 | 6.71 | 6.86 | 6.86 | 6.48 | 6.15 | 6.15 | 5.27 | 5.19 | 5.15 |
| 24 | 5.02 | 3.58 | 2.64 | ≤1.30 | ≤1.30 | ≤1.30 | ≤1.30 | ≤1.30 | ≤1.30 | ≤1.30 | ≤1.30 | ≤1.30 |
| 48 | ≤1.30 | ≤1.30 | ≤1.30 | ≤1.30* | ≤1.30* | ≤1.30* | ≤1.30* | ≤1.30* | ≤1.30* | ≤1.30* | ≤1.30* | ≤1.30* |

**Censored data*

| **Table S5**. Correlation matrix (Pearson) among the diverse variables (independent and dependent) of the data. | | | | | | | | | | | | | | | | | | | | | | | | | | | | | | | | | | | |
| --- | --- | --- | --- | --- | --- | --- | --- | --- | --- | --- | --- | --- | --- | --- | --- | --- | --- | --- | --- | --- | --- | --- | --- | --- | --- | --- | --- | --- | --- | --- | --- | --- | --- | --- | --- |
|  |  |  |  |  |  |  |  |  |  |  |  |  |  |  |  |  |  |  |  |  |  |  |  |  |  |  |  |  |  |  |  |  |  |  |  |
| Variables | Hy | Hy4Glu | Ty | pCum | Verb | HyEda | Ole | Coms | Secox | Seclog | Oleo11 | TPh | Glu | Fru | Sac | Man | TS | pH | Salt | TA | CA | KS | NaB | AA | Cac | LAB | Yeast | 4Dr Ec | 4Dr Sa | 4Dr Lm | 4Dr Se | kmax Ec | kmax Sa | kmax Lm | kmax Se |
| Hy | **1** | **-0.622** | **0.823** | -0.289 | -0.34 | **-0.644** | **-0.675** | **-0.775** | **-0.674** | **-0.728** | **-0.765** | -0.498 | **-0.648** | **-0.649** | **-0.651** | -0.429 | **-0.652** | 0.136 | **-0.822** | 0.035 | 0.271 | **0.666** | **0.716** | 0.527 | 0.414 | 0.002 | -0.022 | -0.173 | -0.447 | -0.346 | **-0.686** | 0.287 | 0.339 | 0.434 | **0.708** |
| Hy4Glu | **-0.622** | **1** | -0.532 | **0.646** | 0.415 | **0.971** | **0.972** | **0.827** | **0.966** | **0.966** | **0.821** | **0.964** | **0.953** | **0.985** | **0.985** | 0.395 | **0.95** | 0.509 | **0.767** | -0.106 | 0.53 | **-0.942** | **-0.895** | **-0.662** | **-0.757** | 0.476 | 0.423 | 0.312 | **0.923** | **0.674** | **0.965** | -0.37 | **-0.83** | **-0.666** | **-0.959** |
| Ty | **0.823** | -0.532 | **1** | -0.313 | -0.52 | -0.466 | -0.524 | **-0.673** | **-0.619** | **-0.632** | **-0.803** | -0.441 | **-0.638** | **-0.587** | -0.561 | **-0.699** | **-0.647** | 0.389 | **-0.834** | -0.331 | 0.17 | **0.635** | **0.729** | 0.217 | 0.074 | 0.237 | 0.222 | -0.245 | -0.455 | -0.538 | -0.508 | 0.351 | 0.41 | **0.628** | 0.516 |
| pCum | -0.289 | **0.646** | -0.313 | **1** | **0.87** | **0.641** | **0.697** | **0.772** | **0.765** | **0.728** | **0.735** | **0.799** | **0.775** | **0.742** | **0.723** | **0.59** | **0.776** | 0.223 | **0.684** | 0.295 | **0.629** | **-0.783** | **-0.772** | -0.223 | -0.305 | 0.125 | 0.185 | **0.8** | **0.782** | **0.743** | **0.624** | **-0.792** | **-0.763** | **-0.744** | **-0.604** |
| Verb | -0.34 | 0.415 | -0.52 | **0.87** | **1** | 0.349 | 0.441 | **0.675** | **0.594** | 0.554 | **0.752** | 0.549 | **0.645** | 0.546 | 0.494 | **0.846** | **0.651** | -0.269 | **0.731** | **0.66** | 0.373 | **-0.652** | **-0.714** | 0.136 | 0.121 | -0.326 | -0.181 | **0.723** | **0.58** | **0.773** | 0.35 | **-0.732** | **-0.611** | **-0.82** | -0.334 |
| HyEda | **-0.644** | **0.971** | -0.466 | **0.641** | 0.349 | **1** | **0.992** | **0.84** | **0.944** | **0.955** | **0.766** | **0.943** | **0.902** | **0.96** | **0.981** | 0.259 | **0.9** | **0.581** | **0.726** | -0.269 | 0.449 | **-0.9** | **-0.843** | **-0.789** | **-0.852** | **0.579** | 0.495 | 0.365 | **0.871** | 0.556 | **0.997** | -0.425 | **-0.754** | -0.547 | **-0.994** |
| Ole | **-0.675** | **0.972** | -0.524 | **0.697** | 0.441 | **0.992** | **1** | **0.896** | **0.973** | **0.981** | **0.833** | **0.96** | **0.939** | **0.98** | **0.992** | 0.362 | **0.938** | 0.5 | **0.796** | -0.166 | 0.453 | **-0.941** | **-0.897** | **-0.729** | **-0.788** | 0.49 | 0.44 | 0.44 | **0.897** | **0.624** | **0.988** | -0.495 | **-0.787** | **-0.625** | **-0.986** |
| Coms | **-0.775** | **0.827** | **-0.673** | **0.772** | **0.675** | **0.84** | **0.896** | **1** | **0.919** | **0.928** | **0.927** | **0.847** | **0.9** | **0.893** | **0.893** | **0.583** | **0.903** | 0.143 | **0.915** | 0.099 | 0.276 | **-0.93** | **-0.935** | -0.532 | -0.518 | 0.15 | 0.183 | **0.649** | **0.8** | **0.663** | **0.847** | **-0.709** | **-0.704** | **-0.713** | **-0.853** |
| Secox | **-0.674** | **0.966** | **-0.619** | **0.765** | **0.594** | **0.944** | **0.973** | **0.919** | **1** | **0.996** | **0.923** | **0.973** | **0.992** | **0.995** | **0.986** | 0.56 | **0.992** | 0.352 | **0.884** | 0.059 | 0.502 | **-0.987** | **-0.968** | -0.564 | **-0.635** | 0.323 | 0.318 | 0.478 | **0.938** | **0.773** | **0.94** | -0.527 | **-0.857** | **-0.783** | **-0.933** |
| Seclog | **-0.728** | **0.966** | **-0.632** | **0.728** | 0.554 | **0.955** | **0.981** | **0.928** | **0.996** | **1** | **0.919** | **0.956** | **0.981** | **0.991** | **0.985** | 0.526 | **0.981** | 0.348 | **0.89** | 0.009 | 0.435 | **-0.979** | **-0.961** | **-0.607** | **-0.666** | 0.333 | 0.328 | 0.446 | **0.913** | **0.729** | **0.955** | -0.503 | **-0.822** | **-0.744** | **-0.952** |
| Oleo11 | **-0.765** | **0.821** | **-0.803** | **0.735** | **0.752** | **0.766** | **0.833** | **0.927** | **0.923** | **0.919** | **1** | **0.829** | **0.942** | **0.891** | **0.857** | **0.799** | **0.946** | -0.032 | **0.988** | 0.355 | 0.312 | **-0.946** | **-0.985** | -0.314 | -0.33 | -0.046 | 0.025 | 0.502 | **0.824** | **0.84** | **0.775** | -0.561 | **-0.775** | **-0.888** | **-0.773** |
| TPh | -0.498 | **0.964** | -0.441 | **0.799** | 0.549 | **0.943** | **0.96** | **0.847** | **0.973** | **0.956** | **0.829** | **1** | **0.963** | **0.98** | **0.975** | 0.46 | **0.961** | 0.518 | **0.766** | -0.001 | **0.665** | **-0.955** | **-0.906** | -0.568 | **-0.688** | 0.442 | 0.417 | 0.489 | **0.964** | **0.758** | **0.926** | -0.516 | **-0.89** | **-0.74** | **-0.913** |
| Glu | **-0.648** | **0.953** | **-0.638** | **0.775** | **0.645** | **0.902** | **0.939** | **0.9** | **0.992** | **0.981** | **0.942** | **0.963** | **1** | **0.986** | **0.963** | **0.64** | **1** | 0.29 | **0.898** | 0.169 | 0.537 | **-0.991** | **-0.981** | -0.466 | -0.554 | 0.239 | 0.257 | 0.456 | **0.947** | **0.835** | **0.897** | -0.501 | **-0.884** | **-0.846** | **-0.888** |
| Fru | **-0.649** | **0.985** | **-0.587** | **0.742** | 0.546 | **0.96** | **0.98** | **0.893** | **0.995** | **0.991** | **0.891** | **0.98** | **0.986** | **1** | **0.994** | 0.504 | **0.985** | 0.416 | **0.844** | 0.002 | 0.526 | **-0.978** | **-0.949** | **-0.603** | **-0.683** | 0.385 | 0.361 | 0.436 | **0.943** | **0.744** | **0.955** | -0.489 | **-0.858** | **-0.747** | **-0.948** |
| Sac | **-0.651** | **0.985** | -0.561 | **0.723** | 0.494 | **0.981** | **0.992** | **0.893** | **0.986** | **0.985** | **0.857** | **0.975** | **0.963** | **0.994** | **1** | 0.422 | **0.962** | 0.471 | **0.81** | -0.095 | 0.499 | **-0.961** | **-0.921** | **-0.681** | **-0.744** | 0.456 | 0.402 | 0.447 | **0.924** | **0.68** | **0.977** | -0.505 | **-0.825** | **-0.68** | **-0.972** |
| Man | -0.429 | 0.395 | **-0.699** | **0.59** | **0.846** | 0.259 | 0.362 | **0.583** | 0.56 | 0.526 | **0.799** | 0.46 | **0.64** | 0.504 | 0.422 | **1** | **0.646** | -0.475 | **0.795** | **0.843** | 0.262 | **-0.63** | **-0.728** | 0.314 | 0.28 | -0.541 | -0.365 | 0.404 | 0.54 | **0.865** | 0.264 | -0.415 | **-0.599** | **-0.92** | -0.25 |
| TS | **-0.652** | **0.95** | **-0.647** | **0.776** | **0.651** | **0.9** | **0.938** | **0.903** | **0.992** | **0.981** | **0.946** | **0.961** | **1** | **0.985** | **0.962** | **0.646** | **1** | 0.279 | **0.903** | 0.174 | 0.529 | **-0.992** | **-0.983** | -0.463 | -0.547 | 0.232 | 0.249 | 0.462 | **0.945** | **0.836** | **0.895** | -0.508 | **-0.883** | **-0.848** | **-0.886** |
| pH | 0.136 | 0.509 | 0.389 | 0.223 | -0.269 | **0.581** | 0.5 | 0.143 | 0.352 | 0.348 | -0.032 | 0.518 | 0.29 | 0.416 | 0.471 | -0.475 | 0.279 | **1** | -0.111 | **-0.679** | **0.589** | -0.277 | -0.126 | **-0.67** | **-0.836** | **0.916** | **0.742** | 0.035 | 0.443 | -0.01 | 0.544 | -0.011 | -0.358 | 0.107 | -0.533 |
| Salt | **-0.822** | **0.767** | **-0.834** | **0.684** | **0.731** | **0.726** | **0.796** | **0.915** | **0.884** | **0.89** | **0.988** | **0.766** | **0.898** | **0.844** | **0.81** | **0.795** | **0.903** | -0.111 | **1** | 0.351 | 0.196 | **-0.902** | **-0.956** | -0.3 | -0.292 | -0.091 | 0.009 | 0.477 | **0.751** | **0.789** | **0.741** | -0.54 | **-0.696** | **-0.849** | **-0.743** |
| TA | 0.035 | -0.106 | -0.331 | 0.295 | **0.66** | -0.269 | -0.166 | 0.099 | 0.059 | 0.009 | 0.355 | -0.001 | 0.169 | 0.002 | -0.095 | **0.843** | 0.174 | **-0.679** | 0.351 | **1** | 0.18 | -0.158 | -0.266 | **0.77** | **0.715** | **-0.803** | **-0.598** | 0.208 | 0.128 | **0.617** | -0.273 | -0.166 | -0.264 | **-0.654** | 0.291 |
| CA | 0.271 | 0.53 | 0.17 | **0.629** | 0.373 | 0.449 | 0.453 | 0.276 | 0.502 | 0.435 | 0.312 | **0.665** | 0.537 | 0.526 | 0.499 | 0.262 | 0.529 | **0.589** | 0.196 | 0.18 | **1** | -0.52 | -0.434 | -0.046 | -0.31 | 0.35 | 0.347 | 0.319 | **0.685** | **0.634** | 0.397 | -0.24 | **-0.709** | -0.543 | -0.366 |
| KS | **0.666** | **-0.942** | **0.635** | **-0.783** | **-0.652** | **-0.9** | **-0.941** | **-0.93** | **-0.987** | **-0.979** | **-0.946** | **-0.955** | **-0.991** | **-0.978** | **-0.961** | **-0.63** | **-0.992** | -0.277 | **-0.902** | -0.158 | -0.52 | **1** | **0.987** | 0.482 | 0.558 | -0.217 | -0.235 | -0.495 | **-0.924** | **-0.82** | **-0.897** | 0.541 | **0.851** | **0.833** | **0.892** |
| NaB | **0.716** | **-0.895** | **0.729** | **-0.772** | **-0.714** | **-0.843** | **-0.897** | **-0.935** | **-0.968** | **-0.961** | **-0.985** | **-0.906** | **-0.981** | **-0.949** | **-0.921** | **-0.728** | **-0.983** | -0.126 | **-0.956** | -0.266 | -0.434 | **0.987** | **1** | 0.397 | 0.446 | -0.088 | -0.136 | -0.502 | **-0.887** | **-0.848** | **-0.846** | 0.555 | **0.826** | **0.877** | **0.841** |
| AA | 0.527 | **-0.662** | 0.217 | -0.223 | 0.136 | **-0.789** | **-0.729** | -0.532 | -0.564 | **-0.607** | -0.314 | -0.568 | -0.466 | **-0.603** | **-0.681** | 0.314 | -0.463 | **-0.67** | -0.3 | **0.77** | -0.046 | 0.482 | 0.397 | **1** | **0.941** | **-0.789** | **-0.596** | -0.175 | -0.439 | 0.053 | **-0.799** | 0.261 | 0.271 | -0.06 | **0.814** |
| CAc | 0.414 | **-0.757** | 0.074 | -0.305 | 0.121 | **-0.852** | **-0.788** | -0.518 | **-0.635** | **-0.666** | -0.33 | **-0.688** | -0.554 | **-0.683** | **-0.744** | 0.28 | -0.547 | **-0.836** | -0.292 | **0.715** | -0.31 | 0.558 | 0.446 | **0.941** | **1** | **-0.855** | **-0.693** | -0.117 | **-0.577** | -0.085 | **-0.844** | 0.169 | 0.43 | 0.048 | **0.85** |
| LAB | 0.002 | 0.476 | 0.237 | 0.125 | -0.326 | **0.579** | 0.49 | 0.15 | 0.323 | 0.333 | -0.046 | 0.442 | 0.239 | 0.385 | 0.456 | -0.541 | 0.232 | **0.916** | -0.091 | **-0.803** | 0.35 | -0.217 | -0.088 | **-0.789** | **-0.855** | **1** | **0.836** | 0.035 | 0.333 | -0.157 | 0.556 | -0.053 | -0.206 | 0.224 | -0.556 |
| Yeast | -0.022 | 0.423 | 0.222 | 0.185 | -0.181 | 0.495 | 0.44 | 0.183 | 0.318 | 0.328 | 0.025 | 0.417 | 0.257 | 0.361 | 0.402 | -0.365 | 0.249 | **0.742** | 0.009 | **-0.598** | 0.347 | -0.235 | -0.136 | **-0.596** | **-0.693** | **0.836** | **1** | 0.132 | 0.251 | -0.107 | 0.457 | -0.139 | -0.098 | 0.137 | -0.478 |
| 4Dr Ec | -0.173 | 0.312 | -0.245 | **0.8** | **0.723** | 0.365 | 0.44 | **0.649** | 0.478 | 0.446 | 0.502 | 0.489 | 0.456 | 0.436 | 0.447 | 0.404 | 0.462 | 0.035 | 0.477 | 0.208 | 0.319 | -0.495 | -0.502 | -0.175 | -0.117 | 0.035 | 0.132 | **1** | 0.451 | 0.39 | 0.347 | **-0.984** | -0.408 | -0.432 | -0.347 |
| 4Dr Sa | -0.447 | **0.923** | -0.455 | **0.782** | **0.58** | **0.871** | **0.897** | **0.8** | **0.938** | **0.913** | **0.824** | **0.964** | **0.947** | **0.943** | **0.924** | 0.54 | **0.945** | 0.443 | **0.751** | 0.128 | **0.685** | **-0.924** | **-0.887** | -0.439 | **-0.577** | 0.333 | 0.251 | 0.451 | **1** | **0.812** | **0.853** | -0.463 | **-0.976** | **-0.797** | **-0.829** |
| 4Dr Lm | -0.346 | **0.674** | -0.538 | **0.743** | **0.773** | 0.556 | **0.624** | **0.663** | **0.773** | **0.729** | **0.84** | **0.758** | **0.835** | **0.744** | **0.68** | **0.865** | **0.836** | -0.01 | **0.789** | **0.617** | **0.634** | **-0.82** | **-0.848** | 0.053 | -0.085 | -0.157 | -0.107 | 0.39 | **0.812** | **1** | 0.546 | -0.383 | **-0.851** | **-0.988** | -0.518 |
| 4Dr Se | **-0.686** | **0.965** | -0.508 | **0.624** | 0.35 | **0.997** | **0.988** | **0.847** | **0.94** | **0.955** | **0.775** | **0.926** | **0.897** | **0.955** | **0.977** | 0.264 | **0.895** | 0.544 | **0.741** | -0.273 | 0.397 | **-0.897** | **-0.846** | **-0.799** | **-0.844** | 0.556 | 0.457 | 0.347 | **0.853** | 0.546 | **1** | -0.415 | **-0.734** | -0.54 | **-0.998** |
| kmax Ec | 0.287 | -0.37 | 0.351 | **-0.792** | **-0.732** | -0.425 | -0.495 | **-0.709** | -0.527 | -0.503 | -0.561 | -0.516 | -0.501 | -0.489 | -0.505 | -0.415 | -0.508 | -0.011 | -0.54 | -0.166 | -0.24 | 0.541 | 0.555 | 0.261 | 0.169 | -0.053 | -0.139 | **-0.984** | -0.463 | -0.383 | -0.415 | **1** | 0.402 | 0.436 | 0.419 |
| kmax Sa | 0.339 | **-0.83** | 0.41 | **-0.763** | **-0.611** | **-0.754** | **-0.787** | **-0.704** | **-0.857** | **-0.822** | **-0.775** | **-0.89** | **-0.884** | **-0.858** | **-0.825** | **-0.599** | **-0.883** | -0.358 | **-0.696** | -0.264 | **-0.709** | **0.851** | **0.826** | 0.271 | 0.43 | -0.206 | -0.098 | -0.408 | **-0.976** | **-0.851** | **-0.734** | 0.402 | **1** | **0.829** | **0.7** |
| kmax Lm | 0.434 | **-0.666** | **0.628** | **-0.744** | **-0.82** | -0.547 | **-0.625** | **-0.713** | **-0.783** | **-0.744** | **-0.888** | **-0.74** | **-0.846** | **-0.747** | **-0.68** | **-0.92** | **-0.848** | 0.107 | **-0.849** | **-0.654** | -0.543 | **0.833** | **0.877** | -0.06 | 0.048 | 0.224 | 0.137 | -0.432 | **-0.797** | **-0.988** | -0.54 | 0.436 | **0.829** | **1** | 0.518 |
| kmax Se | **0.708** | **-0.959** | 0.516 | **-0.604** | -0.334 | **-0.994** | **-0.986** | **-0.853** | **-0.933** | **-0.952** | **-0.773** | **-0.913** | **-0.888** | **-0.948** | **-0.972** | -0.25 | **-0.886** | -0.533 | **-0.743** | 0.291 | -0.366 | **0.892** | **0.841** | **0.814** | **0.85** | -0.556 | -0.478 | -0.347 | **-0.829** | -0.518 | **-0.998** | 0.419 | **0.7** | 0.518 | **1** |

| **Table S6**. Model parameters deduced for the prediction of the inhibition curve biological parameters. | | | | | | | |  |
| --- | --- | --- | --- | --- | --- | --- | --- | --- |
|  |  |  |  |  |  |  |  |  |
| ***Variable*** | ***4Dr Ec*** | ***4Dr Sa*** | ***4Dr Lm*** | ***4Dr Se*** | ***k_max_ Ec*** | ***k_max_ Sa*** | ***k_max_ Lm*** | ***k_max_ Se*** |
| Intercept | -19.75 | -66.274 | -6.015 | 14.222 | 0.966 | 2.414 | 0.993 | 0.526 |
| Hy | 9.965 | 5.447 | -0.674 | -1.846 | -0.205 | -0.095 | 0.002 | 0.041 |
| Hy4Glu | -5.662 | 9.21 | -4.421 | -0.422 | 0.08 | -0.168 | 0.037 | 0.005 |
| Ty | -27.75 | -13.289 | 18.319 | 2.468 | 0.637 | 0.247 | -0.181 | -0.038 |
| pCum | 291.364 | 119.957 | -80.786 | 12.253 | -5.565 | -3.233 | 0.959 | -0.036 |
| Verb | -18.102 | -13.382 | 0.105 | 2.324 | 0.158 | 0.272 | -0.004 | -0.032 |
| HyEda | -37.044 | -36.713 | 39.967 | 16.004 | 0.851 | 0.726 | -0.406 | -0.293 |
| Ole | 3.771 | 3.553 | 0.461 | 0.637 | -0.059 | -0.073 | -0.01 | -0.01 |
| Coms | 675.21 | 454.39 | -330.08 | -129.669 | -15.692 | -6.527 | 2.944 | 1.981 |
| Secox | 1.433 | 2.89 | 0.214 | 0.21 | -0.022 | -0.06 | -0.009 | -0.002 |
| Seclog | 2.973 | 12.949 | 0.379 | 1.278 | -0.024 | -0.279 | -0.03 | -0.016 |
| Oleo11 | 0.127 | -6.007 | 8.224 | 1.792 | 0.007 | 0.151 | -0.112 | -0.041 |
| TPh | 0.784 | 1.651 | 0.02 | 0.008 | -0.015 | -0.032 | -0.003 | 0.001 |
| Glu | -0.124 | 1.866 | -0.296 | -0.022 | 0.002 | -0.038 | 0 | 0.001 |
| Fru | 0.366 | 6.854 | -2.124 | -0.147 | -0.015 | -0.134 | 0.013 | 0.004 |
| Sac | -0.257 | 3.99 | 2.642 | 2.042 | -0.027 | -0.031 | -0.039 | -0.043 |
| Man | 0.385 | 14.674 | 5.135 | -1.298 | 0.004 | -0.31 | -0.12 | 0.045 |
| TS | -0.046 | 1.29 | -0.199 | -0.018 | 0 | -0.026 | 0 | 0.001 |
| pH | 5.973 | 18.531 | -6.821 | -0.832 | -0.105 | -0.39 | 0.069 | 0.025 |
| Salt | -1.055 | -8.053 | 7.976 | 2.397 | 0.07 | 0.142 | -0.093 | -0.044 |
| TA | -4.991 | 24.841 | 6.764 | -5.486 | 0.078 | -0.495 | -0.185 | 0.132 |
| CA | -98.733 | -447.977 | 265.315 | 48.695 | 2.82 | 9.812 | -2.514 | -1.319 |
| KS | 57.414 | 143.119 | -62.386 | -17.818 | -1.117 | -3.194 | 0.574 | 0.478 |
| NaB | 24.887 | 85.179 | -39.089 | -11.922 | -0.536 | -1.866 | 0.384 | 0.309 |
| AA | -56.873 | 107.196 | 19.422 | -18.353 | 1.479 | -2.62 | -0.59 | 0.525 |
| CAc | 15.393 | -25.857 | 25.842 | -5.264 | -0.288 | 0.507 | -0.349 | 0.134 |
| LAB | -1.737 | -0.399 | 2.019 | 0.536 | 0.033 | 0.01 | -0.022 | -0.006 |
| Yeast | 1.328 | -1.655 | -1.726 | -0.488 | -0.029 | 0.044 | 0.02 | 0.003 |
| For the meanings of abbreviation, please consult Table 1 | | | |  |  |  |  |  |

**Figure S1**. HPLC chromatograms of phenolic compounds in table olive brines. The detection was at 280 nm. Peaks: (1) hydroxytyrosol, (2) hydroxytyrosol 4-glucoside, (3) tyrosol, (4) internal standard, syringic acid, (6) *p*-coumaric acid, (7) verbascoside, (8) dialdehydic form of decarboxymethyl elenolic acid linked to hydroxytyrosol (Hy-EDA), (9) oleuropein, (10) secologanoside, (11) comselogoside.


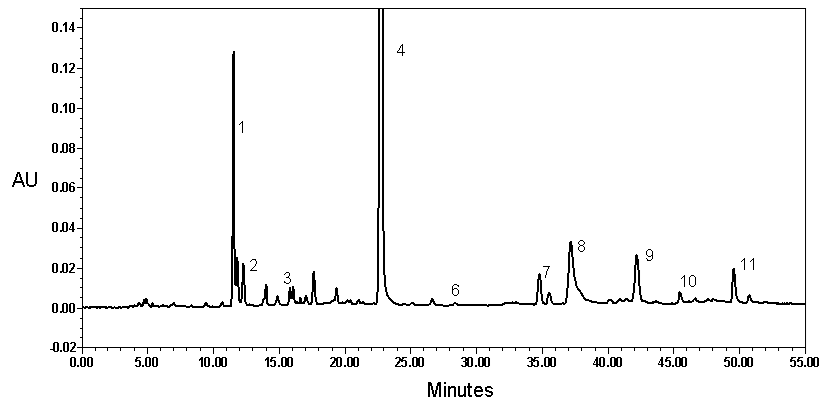


**Figure S2**. HPLC chromatograms of oleosidic compounds in table olive brines. The detection was at 240 nm. Peaks: (1) dialdehydic form of decarboxymethyl elenolic acid (EDA) (2) secoxyloganin, (3) secologanoside, (4) oleoside 11-methyl ester, (5) internal standard, syringic acid.


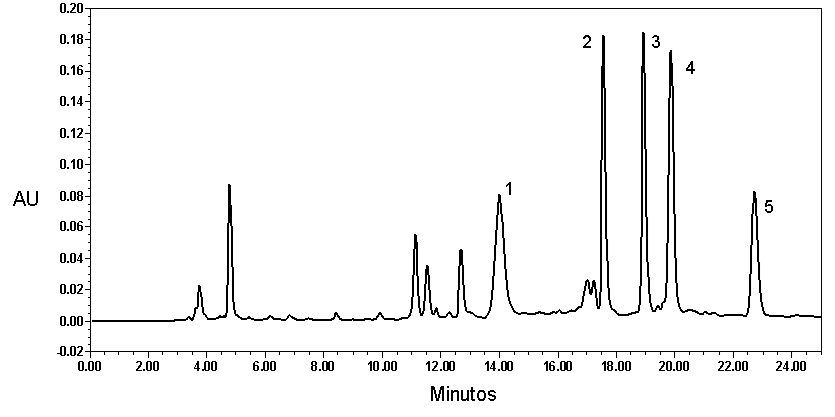


**Figure S3**. HPLC chromatograms of reducing sugars in table olive brines.

**
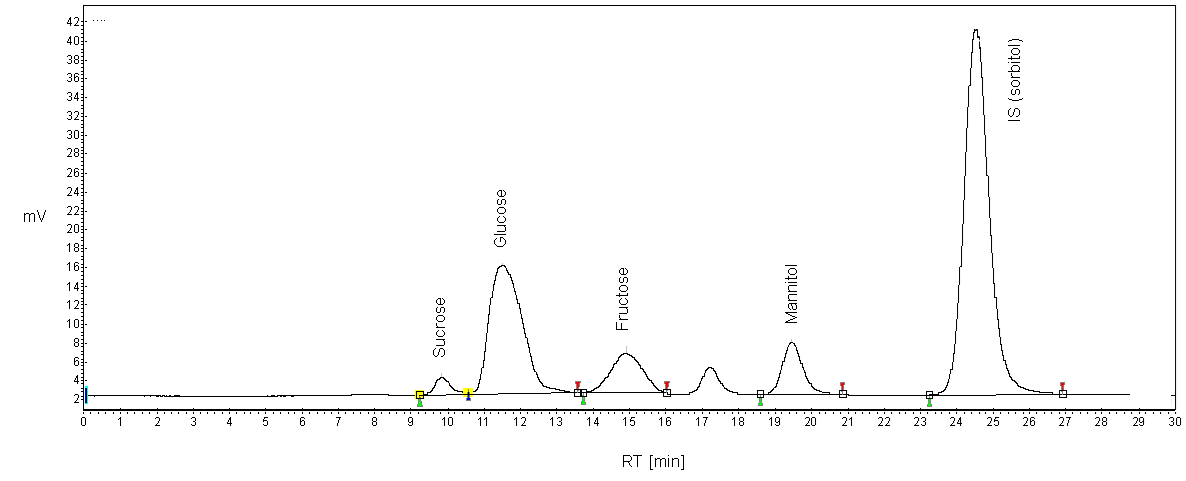
**
